# Large enhancement of the photovoltaic effect in ferroelectric complex oxides through bandgap reduction

**DOI:** 10.1038/srep28313

**Published:** 2016-06-17

**Authors:** Hyunji An, Jun Young Han, Bongjae Kim, Jaesun Song, Sang Yun Jeong, Cesare Franchini, Chung Wung Bark, Sanghan Lee

**Affiliations:** 1 School of Materials Science and Engineering, Gwangju Institute of Science and Technology, Gwangju 61005, South Korea; 2 Department of Electrical Engineering, Gachon University, Seongnam 13120, South Korea; 3 Faculty of Physics and Center for Computational Materials Science, University of Vienna, Vienna A-1090, Austria

## Abstract

Tuning the bandgap in ferroelectric complex oxides is a possible route for improving the photovoltaic activity of materials. Here, we report the realization of this effect in epitaxial thin films of the ferroelectric complex oxide Bi_3.25_La_0.75_Ti_3_O_12_ (BLT) suitably doped by Fe and Co. Our study shows that Co (BLCT) doping and combined Fe, Co (BLFCT) doping lead to a reduction of the bandgap by more than 1 eV compared to undoped BLT, accompanied by a surprisingly more efficient visible light absorption. Both BLCT and BLFCT films can absorb visible light with a wavelength of up to 500 nm while still exhibiting ferroelectricity, whereas undoped BLT only absorbs UV light with a wavelength of less than 350 nm. Correlated with its bandgap reduction, the BLFCT film shows a photocurrent density enhanced by 25 times compared to that of BLT films. Density functional theory calculations indicate that the bandgap contraction is caused by the formation of new energy states below the conduction bands due to intermixed transition metal dopants (Fe, Co) in BLT. This mechanism of tuning the bandgap by simple doping can be applied to other wide-bandgap complex oxides, thereby enabling their use in solar energy conversion or optoelectronic applications.

Solar energy conversion devices based on ferroelectric photovoltaics can potentially exceed the maximum efficiency of conventional p–n junction solar cells^1^,^2^,^3^,^4^,^5^. In ferroelectrics, oriented dipole moments effectively improve the mobility of charge carriers that are generated by light absorption^6^. Despite this advantage, ferroelectrics are not applicable to photovoltaic devices owing to their large energy bandgaps (*E*_*g*_), which lead to insufficient light absorption and consequently limit the photocurrent^7^. Conventional ferroelectric materials (e.g., BaTiO_3_, LiNbO_3_, and Pb(Zr,Ti)O_3_ crystals) exhibit large bandgaps of more than 3 eV. Thus, they can absorb mostly UV light. However, UV light (less than 400 nm) comprises only 3.5% of solar radiation intensity, whereas visible light (400–700 nm) comprises 40% of the solar irradiation^6^. Therefore, to obtain enhanced photocurrents, ferroelectric materials with narrow *E*_*g*_ are necessary for high light absorption including visible light.

The optical bandgap can be adjusted by modulating the composition of ferroelectrics. With the exception of Mott insulators, the wide *E*_*g*_ of ABO_3_-type perovskite ferroelectric materials are governed by the charge transfer from the O 2*p* state to the transition metal (B cation) *d* state^8^. Therefore, most studies on the adjustment of *E*_*g*_ have focused on B-site substitution by a transition metal. A Bi_4_Ti_3_O_12_ (BiT)/LaCoO_3_ (LCO) superlattice thin film is a notable example. The *E*_*g*_ of ferroelectric BiT is decreased from 3.55 to 2.65 eV through the site-specific substitution of the Co ion on the B site of the perovskite octahedral (BO_6_) between the BiT and LCO interfaces^3^. However, this approach may not be preferred for practical photovoltaic applications because fabrication of this material requires the precise control of superlattice periodicity and the use of a complex process with multiple targets. On the other hand, a conventional doping approach is widely used for tuning *E*_*g*_ because of its easy process compared to that required for the fabrication of superlattice thin films.

Based on this point of view, we studied the adjustment of the *E*_*g*_ of a ferroelectric Bi_3.25_La_0.75_Ti_3_O_12_ (BLT) film using a simple doping method based on a theoretical study. Here, we report *E*_*g*_-tuned Co-doped BLT (Bi_3.25_La_0.75_Co_1_Ti_2_O_12_, BLCT) and Fe, Co-doped BLT (Bi_3.25_La_0.75_Fe_0.25_Co_0.75_Ti_2_O_12_, BLFCT) thin films deposited using pulsed laser deposition (PLD). The bandgaps of the BLCT and BLFCT films are lowered to 2.58 and 2.48 eV, corresponding to a bandgap reduction of ~1 eV. In particular, the photocurrent density of BLCT is improved by 6 times relative to that of BLT while maintaining ferroelectricity despite the random doping of the B site in ABO_3_, which is different from the case of the superlattice. Furthermore, the photocurrent density of BLFCT was 25 times higher than that of BLT. The large enhancement of the photocurrent density in BLCT and BLFCT confirms the significant reduction of the bandgaps by simple doping.

## Results and Discussion

### Crystal structure and ferroelectricity

**Figure 1:**
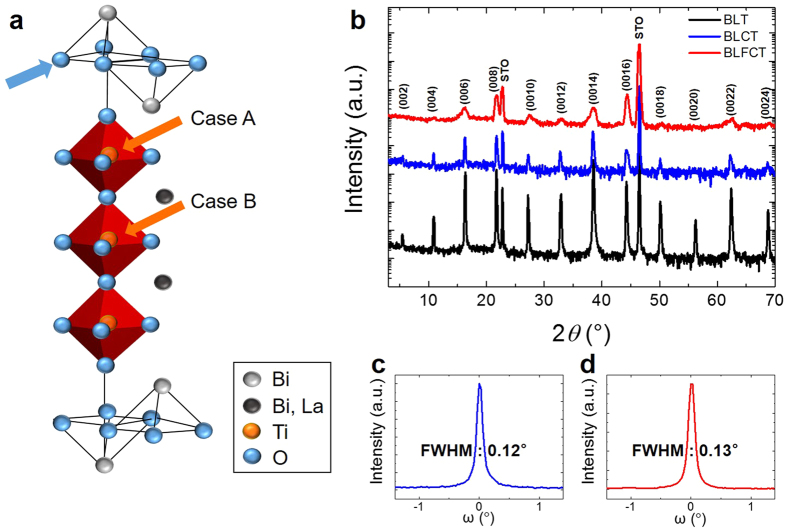
Crystal structures of the BLT, BLCT, and BLFCT films. (**a**) The crystal structure of BLT. The structure shows that La substitutes specific Bi sites of BiT. (**b**) The X-ray *θ*–2*θ* diffraction patterns for BLT, BLCT, and BLFCT epitaxial films on STO (001). The full width at half maximum (FWHM) for the (008) peak in (**c**) the BLCT and (**d**) BLFCT films on STO.

Figure 1(a) shows the half unit cell of BLT^9^. BLT crystalizes in the so-called Aurivillius phase, which consists of the bismuth oxide (Bi_2_O_2_)^2+^ layer and the pseudo-perovskite (Bi_2_Ti_3_O_10_)^2−^ layer containing the TiO_6_ octahedra with La substituting for Bi in the pseudo-perovskite layers. The epitaxial crystalline quality of the grown films was determined by X-ray diffraction (XRD) using a Cu K*α* source (λ = 1.5405 Å). Figure 1(b) shows the *θ*–2*θ* scan of the BLT, BLCT, and BLFCT thin films grown on a (001)-oriented SrTiO_3_ (STO) substrate. The XRD patterns reveal that all films were grown in the 00*l* orientation and that the BLT crystal structure was maintained for the BLCT and BLFCT films. This indicates that Co and Fe doping of BLT does not cause the formation of other phases and that the dopants may be substituted into the BLT structure. Rocking curves for the (008) reflection were measured to determine the out-of-plane mosaic spread and the crystalline quality. As shown in Fig. 1(c,d), the full widths at half maximum (FWHM) of the (008) reflection rocking curves of BLCT and BLFCT are 0.12 and 0.13°, respectively. This indicates that despite the Co and Fe doping, all films exhibit reasonable out-of-plane crystallinity.

**Figure 2:**
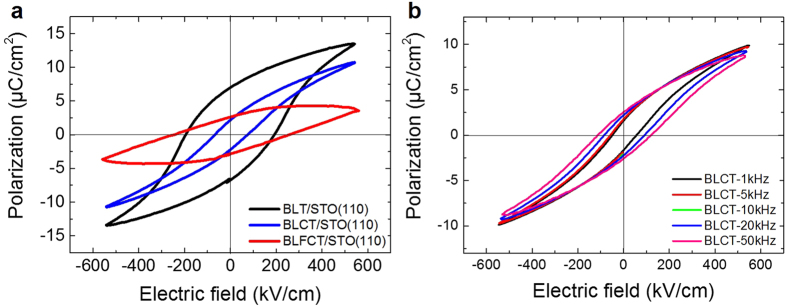
Ferroelectric properties of the BLT, BLCT, and BLFCT films. (**a**) The polarization hysteresis loops were measured using a radiant system at a frequency of 10 kHz for the BLT, BLCT, and BLFCT films on the STO (110) substrates at room temperature. (**b**) The polarization hysteresis loops for the BLCT film measured in the frequency range from 1 to 50 kHz. The loops are maintained at various frequencies.

We measured the polarization (*P*) as a function of the electric field (*E*) of the BLT, BLCT, and BLFCT films, as shown in Fig. 2. The films are deposited on the (110)-oriented STO substrate covered with a SrRuO_3_ bottom electrode for an accurate comparison of ferroelectricity because the polarization orientation of BLT is in-plane and it is difficult to examine the ferroelectricity of the c-axis oriented film^10^. As shown in Fig. 2(a), the BLCT and BLFCT films exhibit ferroelectricity, showing the hysteresis polarization loops, with remanent polarization (*P*_*r*_) values of 2.25 and 2.60 μC/cm^2^ for the BLCT and BLFCT films, respectively. In particular, the BLCT film maintains ferroelectricity at various frequencies, as demonstrated in Fig. 2(b). To summarize, although they may exhibit structural perturbations due to doping, the BLCT and BLFCT films maintain the Aurivillius phase of BLT.

### Optical properties

**Figure 3:**
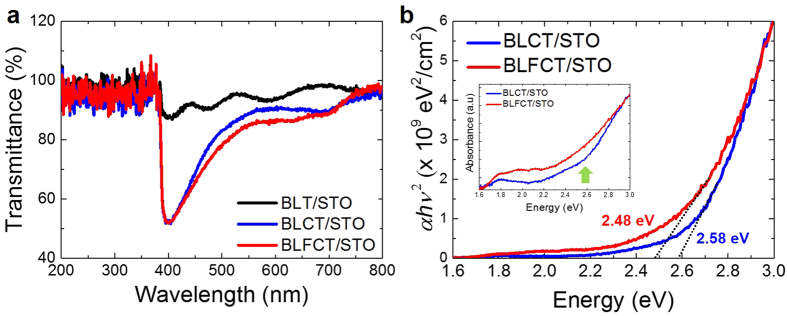
Optical properties of the BLT, BLCT, and BLFCT films. (**a**) The transmittance data depending on the wavelength (200–800 nm) for BLT, BLCT, and BLFCT on the STO (001) substrates. (**b**) The bandgap energy was estimated by extrapolating the linear part of the (αhν)^2^ versus energy plots for BLCT and BLFCT on STO. The inset shows absorbance as a function of energy for BLCT and BLFCT on STO.

The optical properties of the BLT, BLCT, and BLFCT films were measured using an ultraviolet-visible (UV-vis) spectrometer. Figure 3(a) shows the transmittance as a function of the wavelength (200–800 nm) for the BLT, BLCT, and BLFCT films. Inspection of Fig. 3(a) shows that BLCT and BLFCT absorbed visible light in the 380–560 nm wavelength range, whereas BLT scarcely absorbed visible light owing to its large bandgap (3.2–3.8 eV)^11^,^12^. The plots of (*αhν*)^2 ^versus the photo-energy measured for the BLT, BLCT, and BLFCT thin films on STO (001) are shown in Fig. 3(b). The optical bandgaps of the films were estimated using an extrapolation method proposed by Wood and Tauc^13^. The estimated bandgaps of BLCT and BLFCT were 2.58 and 2.48 eV, respectively. Because the transmittance of the films cannot be measured using UV-vis spectroscopy when the *E*_*g*_ of the substrates are lower than those of the films, the transmittances of the BLT films grown on the STO substrate (*E*_*g*_ of STO = 3.2 eV) cannot be obtained, as shown in Fig. 3(a), demonstrating thickness fringes from 380 to 800 nm only. Hence, we also measured the transmittance of the BLT film deposited on a 001-oriented LaAlO_3_ (LAO) substrate because the bandgap of LAO (*E*_*g*_ = 5.6 eV) is much larger than the BLT bandgap. Based on the measured transmittance data of the BLT films on LAO, the *E*_*g*_ was estimated as 3.59 eV, consistent with the reported value (see Supplementary Fig. S1)^11^,^12^. The *E*_*g*_ values of BLCT and BLFCT are respectively, 28% and 31% lower than the experimentally obtained *E*_*g*_ of BLT, as shown in Fig. 3(b).

It is well known that the substitution site is determined by the following two factors: the ionic size and the ionic state. Both Fe and Co are transition metals with 3d orbital states that are identical to that of Ti. Co^2+/3+^ and Fe^2+/3+^ are stable ionic states, and their ionic sizes are smaller than that of Bi^3+^ but are similar to that of Ti^4+^. Because of this similarity in the ionic size, Co and Fe ions may prefer to substitute at the Ti sites in the perovskite blocks rather than at the Bi sites. However, their ionic states differ from that of Ti^4+^. Thus, owing to the mismatch of ionic states, it is not certain that Co and Fe ions can replace Ti ions. However, Choi *et al*. confirmed that Co can replace Ti in a BiT/LCO superlattice film, using both the experimental result and density functional theory (DFT) electronic calculation^3^. Fe could also substitute for Ti in the perovskite blocks randomly, as was verified in the case of the fabrication of a Bi_4_Ti_3_O_12_ film doped with LaFeO_3_^14^,^15^. Consistent with these previously reported results, Co and Fe ions appear to replace Ti ions at the perovskite blocks of BLT in our experiment, as we confirmed that the BLCT and BLFCT bandgaps are narrower than that of the BLT film.

Many studies have been performed on bandgap tuning of BiT thin films by transition metal doping^16^,^17^. Among these, the bandgap of a BiT-LCO superlattice film was significantly reduced, confirming that Co ions can be the most appropriate dopants^16^. In the case of the BiT-LCO superlattice, the bandgap could be reduced because of the occurrence of Co ion substitution for the Ti ions in the (outer) perovskite blocks near the Bi_2_O_2 _layer owing to the interface interaction between the BiT and LCO layers^3^. We expected that not only site-specific substitution in the superlattice but also random intermixing of Co by doping could decrease the bandgap while maintaining the ferroelectricity. The optical bandgap of BLCT, 2.58 eV, is similar to the bandgap (2.65 eV) of the superlattice film. Consequently, the bandgap of BLT can be tuned using the intermixing approach, which is much simpler than the site-specific substitution approach.

Furthermore, the bandgap of the BLFCT film decreased more than that of BLCT. Fe and Co dopants are expected to be located randomly at the Ti sites in perovskite blocks based on their mixing ratio (1:3) due to their similar ionic sizes and stable ionic states. Therefore, additional doping of Fe may reduce the bandgap of BLT more than that of Co.

### Electronic structure

To reveal the mechanism of the bandgap reduction upon doping, we analysed the electronic structure using DFT calculations. Because of the mismatch between the oxidation states of Ti (4+) and doped ions (+2/+3 for Co and Fe), oxygen vacancies occur upon doping. We found that the most probable position of an oxygen vacancy was between the Bi_2_Ti_3_O_10_ perovskite blocks (indicated by the blue arrow in Fig. 1(a)) by more than 140 meV per formula unit compared to other possible positions, consistent with previous studies^18^,^19^.

In addition to the position of the oxygen vacancy, the position of the substitutional site is an important factor. The previous site-specific doping approach uses the superlattice structure to ensure that the dopant ion replaces a Ti ion at the outer position (case A) of a perovskite block (see Fig. 1(a))^3^. In our simpler intermixing approach, however, doped ions can be located on the inner site (case B) of a perovskite block. When no oxygen vacancies exist, doped ions prefer case B to case A. However, because the 4+ charge states are unlikely for both Fe and Co, the gap reduction was found to be highly dependent on the dopant position. By introducing oxygen vacancies, the much more stable 3+ charge state can be obtained for both Co and Fe with enhanced robustness of the gap size against different configurations of the dopant positions.

**Figure 4:**
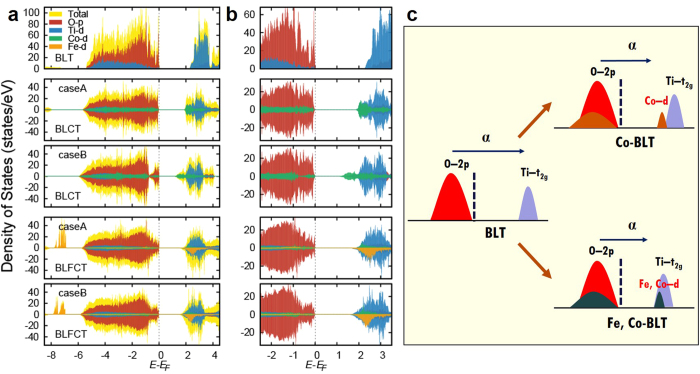
Electronic structures of the BLT, BLCT, and BLFCT films. (**a**) The DOS of each film. For the BLCT and BLFCT cases, cases A and B are shown. The bandgap is governed by the highest occupied O-*p* and lowest unoccupied transition metal *d* levels. (**b**) Magnification of the DOS near the Fermi level. The lowest unoccupied states are determined mostly by doped Co- and Fe*-d* states. (**c**) The effect of doping on the overall electronic levels. The bandgap is due to the *d*-levels of the doped transition metal ions.

In Fig. 4(a,b), we show the density of states (DOS) of all three cases: undoped BLT, BLCT, and BLFCT. In the second and third cases, we included oxygen vacancies for both configurations of dopant ions (cases A and B). Inspection of the DOS of the pristine BLT shows that as expected, the overall bandgap is determined mostly by the occupied O-*p* level and unoccupied Ti-*d* level. The calculated bandgap size is 2.22 eV, somewhat smaller than the experimental value due to usual underestimation within the DFT-based approach but consistent with previous calculations^3^. When BLT is doped by Co (BLCT), clear reductions of the bandgaps by 1.86 and 1.04 eV for cases A and B, respectively, can be seen. This is due to the energy levels of the unoccupied Co-*d* states. Doped Co^3+^ has a nonmagnetic phase with *t*_*2g*_^6^ electronic configurations where the unoccupied *e*_*g*_ level is located lower than the Ti-*d* levels of undoped BLT. In cases A and B, the reductions of the gap are 16% (from 2.22 to 1.86 eV) and 53% (from 2.22 to 1.04 eV), respectively. We assume that the position of the dopant ion is important for the effective reduction of the bandgap. Because the experimental result shows that the reduction is approximately 28% (from 3.59 to 2.58 eV), we assume that the intermixing approach results in a mixture of different configurations including cases A and B. The overall schematic diagram of the doping effects is shown in Fig. 4(c). By comparing the DOSs for cases A and B, we find that the location of the unoccupied Co-*d* states, which determine the magnitude of the *E*_*g*_ of BLCT, is highly dependent on the geometric position of the dopant Co ion within a perovskite block. While case B shows a split-off of Co-*d* (*e*_*g*_) states from Ti-*d* states to form mid-gap-like electronic states, case A shows large hybridization of Co-*d* and Ti-*d* without the mid-gap phase, consistent with a recent optical conductivity study on the supercell structure where no split-off Co-*d* states are found^20^.

In our UV-vis data (the inset in Fig. 3(b)), we can see the shoulder region near 2.6 eV, which was absent for pristine BLT (see Fig. S1). We can relate this to the Co-*e*_*g*_ states that are located closer to the Fermi level (Fig. 4(b)). The energetics shows that case B is more stable than case A with a difference of 116 meV/f.u.; however, due to the sample fabrication process at a high temperature, two and more phases can mix, as we can conclude from the previous analysis of the bandgap data. Although the number of oxygen vacancies is not defined, considering that the 2+ and 3+charge states can both effectively reduce *E*_*g*_^3^, Co doping is an efficient method for tuning the optical gap of a BLT system. We also investigated the effect of additional Fe doping (BLFCT), as described before. The calculated bandgaps for cases A and B are 1.56 and 1.51 eV, respectively; these values are close to each other and are consistent with the experimentally observed bandgap reduction of 31%.

From the DOSs of both configurations (the fourth and fifth panels in Fig. 4(a,b), respectively), we can observe that the overall energy levels of the transition metal ions do not change significantly. Fe has a 3+ charge state with high-spin configurations (*t*_*2g↑*_^*3*^*e*_*g↑*_^*2*^) where unoccupied *d*-states are well hybridized with Ti- and Co-*d* orbitals without forming the mid-gap state, as schematically shown in Fig. 4(c). This is also consistent with our UV-Vis data shown in the inset of Fig. 3(b).

In the case of BLCT, the curve shows a steep slope starting from 3.0 eV, which is suddenly flattened near the shoulder position (2.6 eV), suggesting the presence of mid-gap states. In the case of BLFCT, no such shoulder or peak is observed, with the overall form of the curve exhibiting a more gradual character than that in the case of BLCT. In general, the *t*_*2g*_–*t*_*2g*_ orbital hopping integrals are larger than those for the *t*_*2g*_–*e*_*g*_ orbitals^21^, providing better hybridization for BLFCT than for BLCT. The electronic structures for cases A and B are very similar for BLFCT compared to BLCT, suggesting that the BLFCT sample is more robust against the disorder of the dopant ions than BLCT. Further studies using different dopants with various electronic states are needed to elucidate the microscopic mechanism of the gap reduction.

### Photocurrent response

**Figure 5:**
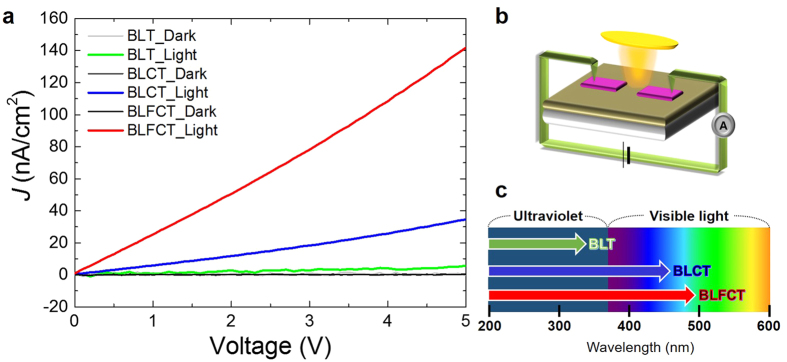
Photovoltaic responses of the BLT, BLCT, and BLFCT films. (**a**) The photocurrent density (*J*) of the BLT, BLCT, and BLFCT films on STO (001). The dark current of the BLT, BLCT, and BLFCT films (grey, dark grey, and black). The light current of the BLT film (green). The light current of the BLCT film (blue). The light current of the BLFCT film (red). (**b**) The schematic of the photocurrent measurement. The ITO electrodes were deposited on the films, and *J* was measured between the two electrodes. (**c**) The schematic of the light wavelength range that the BLT, BLCT, and BLFCT films can absorb, which is predicted using the bandgap data.

The photocurrent density (*J)* was measured to confirm the effect of narrowing the bandgaps of the BLCT and BLFCT films. As shown in Fig. 5(a), the BLT, BLCT, and BLFCT films exhibited nearly zero current without light irradiation (dark current), while their photocurrent responses under light irradiation were clearly enhanced. Compared to the *J* of BLT (5.71 nA/cm^2^), the *J* of BLCT (34.58 nA/cm^2^) was improved 6 times and the *J* of BLFCT (141.92 nA/cm^2^) was improved 25 times, as shown in Fig. 5(a,c). We note that a transparent conducting oxide, indium tin oxide (ITO), was used as the electrode on the film to maximize the light-irradiated region. The photocurrent was measured between the ITO electrodes on the films at a voltage from 0 to 5 V under the light field, as shown Fig. 5(b).

These results confirm that the bandgap narrowed by doping also improves the photocurrent density of BLT. Conclusively, this indicates that the BLCT and BLFCT films have enhanced optical properties and can absorb more photons effectively. Therefore, they can be applied to various devices such as photovoltaics.

## Conclusion

Here, we successfully narrowed the bandgap of BLT by more than 1 eV while maintaining ferroelectricity through a simple Fe, Co-doping approach. The results of the experiments conducted using a Co-doped BLT (BLCT) film show that the simpler intermixing approach, and not only the site-specific approach, can tune the bandgap of BLT. We also observed that additional Fe doping contributed to the decrease in the bandgap of BLT and improved the photocurrent density by 25 times relative to that of BLT. This approach can be used as a method for tuning the bandgaps of a variety of perovskite ferroelectric films by examining their substitution mechanisms and dopant effect on the decrease in their bandgaps. These results indicate that ferroelectrics with large bandgaps can generally be more easily applied for use in photovoltaic devices.

## Methods

### Sample fabrication

All targets for the film depositions were synthesized using a solid-state reaction method with the following starting binary oxide powders as raw materials: Bi_2_O_3_ (99.9%, Kojundo Chemical Co. Ltd., Japan), TiO_2_ (99.99%, Kojundo Chemical Co. Ltd., Japan), La_2_O_3_ (99.99%, Kojundo Chemical Co. Ltd., Japan), Co_3_O_4_ (99.99%, Kojundo Chemical Co. Ltd., Japan), and Fe_2_O_3_ (99.9%, Kojundo Chemical Co. Ltd., Japan). Based on our previous results, the doping concentrations of Co and Fe atoms in the doped BLT targets were chosen to maximize the bandgap reduction, and the doping ratios were Bi_3.25_La_0.75_Co_1_Ti_2_O_12_ (BLCT) and Bi_3.25_La_0.75_Fe_0.25_Ti_2_O_12_ (BLFCT)^22^,^23^. The BLT, BLCT, and BLFCT powders were blended thoroughly at the stoichiometric ratio in a ball mill for 24 h, dried in an oven at a temperature of 100 °C, and calcined at 700 °C for 2.5 h in air. The powders were pressed for 5 min at a pressure of 50 MPa and then sintered at 950 °C for 2.5 h.

The BLT, BLCT, and BLFCT epitaxial thin films (thickness of 250 nm) were fabricated using pulsed laser deposition (KrF excimer laser, λ = 248 nm), operating at a repetition rate of 10 Hz, on SrTiO_3_ (001) substrates under 80 sccm O_2_ gas flow. The operating temperature for the BLT, BLCT, and BLFCT films was 750 °C, and the laser energy density for these films was 2.0 J/cm^2^. In particular, the films were deposited at a high frequency of 10 Hz to prevent the loss of the volatile Bi.

### Thin film characterization

The structural properties were analysed by XRD using Cu Kα radiation (X’pert PRO, PANalytical, Netherlands). The polarization hysteresis loop was measured by a ferroelectric test system (Precision Multiferroic, Radiant Technologies, Inc., USA). For the polarization hysteresis loop measurement, the BLT, BLCT, and BLFCT films were deposited on a SrRuO_3_ bottom electrode covered with STO (110) substrates. Then, a Pt top electrode (diameter of 150 μm) was deposited on the films using RF sputtering. The polarization loops of the films were measured in the c-plane orientation between the bottom electrode (SrRuO_3_) and the top electrode (Pt). The optical properties were analysed using an ultraviolet-visible (UV-Vis) NIR absorption spectrometer (Cary500Scan, Varian, Australia). The optical properties of the films were measured under light with wavelengths ranging from 200 to 800 nm. We use same thickness for all three films (pristine BLT, BLCT, BLFCT) to avoid the absorption coefficient shift due to the thickness effect^24^. The photocurrent density was measured using a sourcemeter (SourceMeter 2410, Keithley, United States). The measurement was implemented under light illumination using a 100 W solar simulator (K3400, McScience, Korea). ITO transparent electrodes were deposited on the surface of the films with a separation of 100 μm using DC sputtering. The photocurrent densities of the films were measured between the ITO transparent electrodes after contacting two tips with the respective electrodes.

### Density functional calculations

Electronic structure calculations were performed using the projector-augmented-wave method implemented in the Vienna ab initio simulation package (VASP)^25^,^26^. We adopted the generalized gradient approximation (GGA) functional in the Perdew–Burke–Ernzerhof parameterization including an on-site Coulomb parameter U = 5 eV for the *d*-orbitals of both Co and Fe within the Dudarev approach^27^,^28^, based on previous studies in the literature on similar systems^3^,^29^. We employed supercells containing 4 formula units: Bi_16_Ti_12_O_48_ for BLT, Bi_16_Ti_8_Co_4_O_46_ for BLCT and Bi_16_Ti_8_Fe_2_Co_2_O_46_ for BLFCT. Lattice parameters were fixed to the experimental values, and all internal atomic positions were fully relaxed for all cases. A plane-wave cut-off of 400 eV was used with a 4 × 4 × 1 Monkhorst–Pack k-point sampling mesh.

## Additional Information

**How to cite this article**: An, H. *et al*. Large enhancement of the photovoltaic effect in ferroelectric complex oxides through bandgap reduction. *Sci. Rep*. **6**, 28313; doi: 10.1038/srep28313 (2016).

## Supplementary Material

Supplementary Information
