# Supplementary material for: Large enhancement of the photovoltaic effect in ferroelectric complex oxides through bandgap reduction
Source: Sci Rep. 2016 Jun 17;6:28313. doi: 10.1038/srep28313 (PMC4911611; doi:10.1038/srep28313)
Supplement: Supplementary Information [file srep28313-s1.doc]

Supplementary information

**Large enhancement of the photovoltaic effect in ferroelectric complex oxides through bandgap reduction**

Hyunji An^1^, Jun Young Han^2^, Bongjae Kim^3^, Jaesun Song^1^, Sang Yun Jeong^1^ Cesare Franchini^3^, Chung Wung Bark^2*^& Sanghan Lee^1*^

^1^School of Material Science and Engineering, Gwangju Institute of Science and Technology, Gwangju 61005, South Korea

^2^Department of Electrical Engineering, Gachon University, Seongnam 13120, South Korea

^3^Faculty of Physics and Center for Computational Materials Science, University of Vienna, Vienna A-1090, Austria

Correspondence and requests for materials should be addressed to C. W. Bark ([bark@gachon.ac.kr](mailto:bark@gachon.ac.kr)) and S. Lee ([sanghan@gist.ac.kr](mailto:sanghan@gist.ac.kr))

**S1. Verification of the bandgap of the BLT epitaxial thin film.**

We fabricated a BLT film on an LAO (001) substrate to verify the bandgap of BLT, which could not be observed in the film on the STO (001) substrate. After the film fabrication, the structural and optical properties of the film were demonstrated using XRD and a UV-vis spectrometer, respectively. The bandgap of BLT on LAO measured using the UV-vis spectrometer was consistent with the reported value.


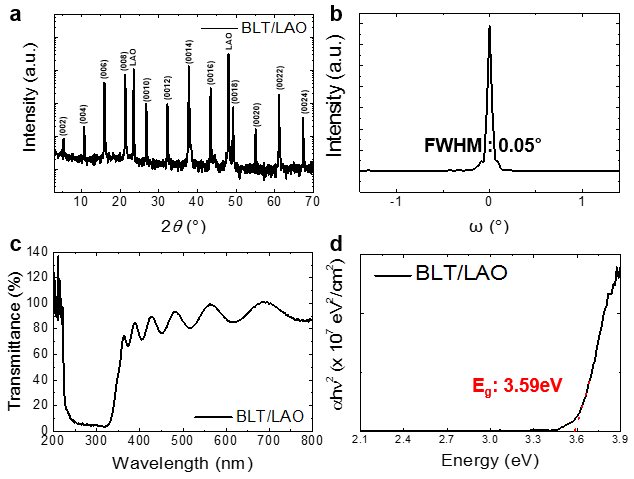


**Figure S1** | (a) X-ray *θ*–2*θ* diffraction pattern for the BLT film on LAO (001). (b) The FWHM of (008) peak of the BLT film on LAO. (c) The transmittance data depending of the wavelength for the BLT film on LAO measured using a UV-vis spectrometer. (d) The bandgap energy was estimated extrapolating the linear part of the (αhν)^2^ versus energy plots for the BLT film on LAO.

**S2. Calculation of dielectric function**

We have calculated the dielectric function of each BLT, and doped cases. In accordance with DOS calculation data, we can clearly observe the band gap is reduced for BLCT and BLFCT cases. Each BLCT and BLFCT cases are for the ground phase, respectively.


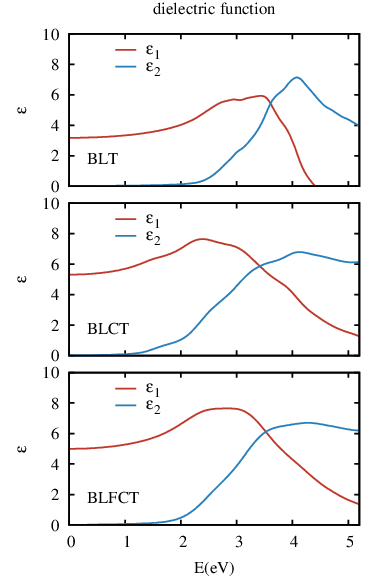


**Figure S2** | The calculated real (ε_1_) and imaginary (ε_2_) part of the dielectric functions for BLT, BLCT, and BLFCT.
